# Compensatory hyperhidrosis following microwave-based therapy for axillary hyperhidrosis

**DOI:** 10.1016/j.jdcr.2026.02.046

**Published:** 2026-03-06

**Authors:** Davin Lim, Xiaozhun (Chloe) Hang

**Affiliations:** a Cutis Clinic, Indooroopilly, Queensland, Australia; b Mowbray Park Medical & Dermatology Clinic, East Brisbane, Queensland, Australia

**Keywords:** axillary hyperhidrosis, compensatory hyperhidrosis, microwave ablation, microwave-based therapy complication

## Introduction

Primary axillary hyperhidrosis is a chronic condition marked by sweating that clearly exceeds thermoregulatory needs and, in many patients, becomes socially and professionally disabling. In day-to-day practice, management usually begins with topical antiperspirants, topical antimuscarinic agents (such as glycopyrronium tosylate or sofpironium bromide), iontophoresis, oral anticholinergic agents, or intradermal botulinum toxin injections.^1^ These options can be effective, but their limitations are well recognized, including incomplete response, systemic side effects, and the need for ongoing repeat treatments.

Microwave-based therapy (miraDry; Sientra) received US Food and Drug Administration approval in 2011 as a medical device for the nonsurgical treatment of axillary hyperhidrosis. The device delivers focused microwave energy to the dermal-subcutaneous junction, where eccrine and apocrine glands reside, creating localized thermal injury while protecting the epidermis through active cooling. As sweat glands do not regenerate, the effect is generally considered permanent. Published studies report mean reductions in axillary sweating and odor of around 80% to 90% after 1 or 2 sessions.

Compensatory hyperhidrosis is a well-recognized consequence of endoscopic thoracic sympathectomy, yet it has not been described in association with microwave-based axillary therapy as a documented clinical adverse event in the published literature. Here, we describe a case of new-onset persistent nonaxillary hyperhidrosis that appeared shortly after microwave-based therapy and remained stable over long-term follow-up.

## Case report

A 29-year-old man was referred by his general practitioner for treatment of axillary hyperhidrosis. He had previously declined intradermal botulinum toxin injections recommended by another dermatologist, citing both the need for repeat treatments and a preference for a more permanent solution. He was otherwise healthy, took no regular medications, and reported no personal or family history of hyperhidrosis or autonomic dysfunction.

Clinical assessment was consistent with primary axillary hyperhidrosis, with no features to suggest a secondary cause. After a discussion of available options, including their benefits and limitations, the patient elected to proceed with microwave-based therapy. Written informed consent was obtained from the patient for publication of this case report, covering commonly discussed risks such as infection, burns, paresthesia, and altered sensation, including theoretical risks listed by the manufacturer, such as compensatory hyperhidrosis. Treatment was performed using medium-level energy settings in line with standard protocols.

Approximately 12 days after the procedure, the patient contacted the clinic to report new-onset excessive sweating outside the axillae, most noticeable over the lumbosacral region. He described sweating during both daytime activities and sleep. At the same time, he noted only minimal improvement in axillary sweating.

Clinical examination corroborated the patient’s history, demonstrating persistent axillary hyperhidrosis and new-onset truncal sweating. At rest in a temperature-controlled clinic environment, visible beads of sweat were observed over the lower thoracolumbar region, extending bilaterally from T10 to L3 dermatomes, while surrounding skin appeared otherwise normal. No prior history of truncal hyperhidrosis was reported, and the distribution was distinct from the axillary treatment zones.

Oral propantheline bromide was commenced at a dose of 15 mg twice daily, resulting in partial symptom relief. Follow-up reviews were conducted at 6, 18, and 30 months. Over this period, both axillary and nonaxillary sweating persisted, without clear improvement or progression. Referral for a vascular surgical opinion regarding endoscopic thoracic sympathectomy was discussed but ultimately declined by the patient. At last review, symptoms remained stable.

## Discussion

Microwave-based axillary therapy is now widely used for primary axillary hyperhidrosis and is generally associated with high patient satisfaction. Larger case series and longer-term follow-up studies tend to describe adverse effects that are local and transient, such as swelling, tenderness, induration, and temporary sensory disturbance.^2^, ^3^, ^4^ In routine practice, these effects are usually self-limiting and resolve without intervention.

More serious complications have been described, albeit rarely. Documented events include brachial plexus and peripheral nerve injuries,^5^ and deep thermal injuries and burns requiring specialist wound care have also been described.^6^ Postmarket surveillance data from the Manufacturer and User Facility Device Experience database highlight reports of neurological symptoms, infections, and burns, reminding clinicians that uncommon adverse events may not be fully captured in preapproval trials.^7^ A fatal case of necrotizing fasciitis complicated by streptococcal toxic shock syndrome following microwave-based therapy has also been reported.^8^ Notably, this occurred after off-label treatment of the perineal region and highlighted the potential severity, if rarity, of serious complications. Table I summarizes reported adverse events associated with microwave-based therapy in the published literature.

**Table I:** Reported adverse events associated with microwave-based therapy in the published literature

Category	Adverse event	Summary	References	Year
Common (transient)	Swelling, pain, erythema, bruising	Expected local reactions, typically resolve over weeks-months	Wang et al, Wimmer et al^2^^,^^9^	2023, 2025
	Induration/nodularity	Temporary subcutaneous firmness	Scuderi et al, Lin et al^4^^,^^10^	2017, 2021
	Temporary sensory change	Numbness/paresthesia, usually self-limited	Lin et al, Wang et al, Wimmer et al^2^^,^^4^^,^^9^	2021, 2023, 2025
Neurologic (rare)	Brachial plexus injury (bilateral)	Motor/sensory deficits, incomplete recovery reported	Puffer et al^5^	2019
	Median nerve-predominant plexopathy	Likely traction/positioning, conservative recovery	Lee et al^11^	2021
Thermal/tissue injury (rare)	Thermal burn/necrosis	Full-thickness soft-tissue injury requiring burn care	Bayoux et al, Albucker et al^6^^,^^7^	2022, 2023
Infectious (very rare)	Soft-tissue infection	Some requiring antibiotics/surgery	Albucker et al^7^	2023
	Necrotizing fasciitis, STSS, death∗	Rapidly progressive infection	Wen et al^8^	2022

*STSS*, Streptococcal toxic shock syndrome.

∗ This fatal case occurred following off-label microwave-based treatment of the genital and perineal regions rather than standard axillary use.

By contrast, compensatory hyperhidrosis has not been documented in clinical trials, long-term cohort studies, patient surveys, or postmarket surveillance reports of microwave-based axillary therapy^2^, ^3^, ^4^^,^^9^^,^^10^ despite being listed by the manufacturer as a theoretical adverse effect in patient consent materials.

This case report describes a compensatory-like, nonclassical pattern of hyperhidrosis with onset shortly after microwave-based axillary therapy and stability over extended follow-up. While a single case cannot establish causation, the timing and distribution of symptoms may suggest an association in this individual.

Importantly, this presentation is unlikely to represent classical compensatory hyperhidrosis as described following endoscopic thoracic sympathectomy, particularly given the persistence of axillary sweating. Instead, we propose a mechanism of autonomic dysregulation and functional sympathetic adaptation following incomplete or heterogeneous injury to axillary eccrine glands and their associated sudomotor innervation. Partial disruption of peripheral autonomic feedback, without complete denervation, may alter central thermoregulatory signaling^12^ and result in increased sudomotor output in anatomically distinct but sympathetically linked regions, such as the thoracolumbar dermatomes. This mechanism does not require complete abolition of sweating at the treated site and may, in this context, explain the coexistence of persistent axillary hyperhidrosis and new-onset truncal sweating observed in this patient. An additional consideration is the unmasking of subclinical generalized hyperhidrosis, whereby baseline autonomic hyperexcitability becomes clinically apparent following intervention.

In conclusion, this case does not imply that compensatory hyperhidrosis is a common or expected outcome of microwave-based therapy. Rather, it broadens the spectrum of observed patient experiences and serves as a reminder that unexpected patterns can emerge outside controlled study settings. Awareness of this potential outcome may assist clinicians in counseling patients considering this treatment and highlights the value of continued postmarket surveillance.

### Declaration of generative AI and AI-assisted technologies in the writing process

No generative AI or AI-assisted technologies were used in the preparation of this manuscript.

## Conflicts of interest

None disclosed.
